# Transcriptional Regulation of the Outer Membrane Porin Gene *ompW* Reveals its Physiological Role during the Transition from the Aerobic to the Anaerobic Lifestyle of *Escherichia coli*

**DOI:** 10.3389/fmicb.2016.00799

**Published:** 2016-05-31

**Authors:** Minfeng Xiao, Yong Lai, Jian Sun, Guanhua Chen, Aixin Yan

**Affiliations:** ^1^School of Biological Sciences, The University of Hong KongHong Kong, China; ^2^Department of Chemistry, The University of Hong KongHong Kong, China

**Keywords:** outer-membrane porin protein, anaerobic adaptation, transcription regulation, molecular docking, colicin mediated killing

## Abstract

Understanding bacterial physiology relies on elucidating the regulatory mechanisms and cellular functions of those differentially expressed genes in response to environmental changes. A widespread Gram-negative bacterial outer membrane protein OmpW has been implicated in the adaptation to stresses in various species. It is recently found to be present in the regulon of the global anaerobic transcription factor FNR and ArcA in *Escherichia coli*. However, little is known about the physiological implications of this regulatory disposition. In this study, we demonstrate that transcription of *ompW* is indeed mediated by a series of global regulators involved in the anaerobiosis of *E. coli*. We show that FNR can both activate and repress the expression of *ompW* through its direct binding to two distinctive sites, -81.5 and -126.5 bp respectively, on *ompW* promoter. ArcA also participates in repression of *ompW* under anaerobic condition, but in an FNR dependent manner. Additionally, *ompW* is also subject to the regulation by CRP and NarL which senses the availability and types of carbon sources and respiration electron acceptors in the environment respectively, implying a role of OmpW in the carbon and energy metabolism of *E. coli* during its anaerobic adaptation. Molecular docking reveals that OmpW can bind fumarate, an alternative electron acceptor in anaerobic respiration, with sufficient affinity. Moreover, supplement of fumarate or succinate which belongs to the C_4_-dicarboxylates family of metabolite, to *E. coli* culture rescues OmpW-mediated colicin S4 killing. Taken together, we propose that OmpW is involved in anaerobic carbon and energy metabolism to mediate the transition from aerobic to anaerobic lifestyle in *E. coli*.

## Introduction

Carbon and energy homeostasis is essential for bacterial physiology and survival in a constantly changing environment. Maintenance of this homeostasis is often achieved by coordinated regulatory networks that involve complex signal transduction pathways and gene expression changes. Anaerobiosis, a predominant physiological adaptation undergone by enteric bacteria during their transition to the host gastrointestinal tract, is mediated primarily by the coordinated action of two global transcription regulatory systems: Fumarate and Nitrate Reduction (FNR) and Aerobic respiratory control (ArcAB) (Green and Paget, 2004; Fleischhacker and Kiley, 2011). While the single component transcription regulator FNR directly senses the absence of molecular O_2_ in the cytoplasm of bacteria and activates the expression of genes important to the anaerobic lifestyle of the bacterial species, the two component system ArcAB senses the redox status in the cytoplasmic membrane of bacteria through its membrane sensor ArcB, and upon activated, primarily represses the expression of genes involved in the aerobic carbon oxidation through its cognate response regulator ArcA (Green and Paget, 2004; Fleischhacker and Kiley, 2011; Myers et al., 2013; Park et al., 2013). In addition to the enzymes of central metabolic pathways, several recent genome-wide studies have revealed that FNR and ArcAB also regulate a large number of genes outside the physiological processes of carbon and energy metabolism, including the genes involved in motility, virulence, membrane structures, as well as those with unknown functions (Salmon et al., 2003; Kang et al., 2005; Salmon et al., 2005; Constantinidou et al., 2006; Grainger et al., 2007; Evans et al., 2011; Myers et al., 2013; Park et al., 2013). Of particular interest is a wide spread outer membrane protein W (OmpW), which has been found to be within the core regulon of FNR and is also regulated by ArcAB in *E. coli* (Dufour et al., 2010; Myers et al., 2013; Park et al., 2013). However, the cellular functions of OmpW and its physiological relevance to bacterial anaerobiosis are not known.

The outer membrane (OM) of Gram-negative bacteria provides as a permeability barrier that hinders the entry of both toxic molecules and nutrients. To facilitate the selective entry of nutrients and other molecules that are necessary for the growth and function of the cell, Gram-negative bacteria use protein channels called porins within their OM. In addition to the major porin proteins which form trimeric, hydrophilic barrels composed of 12-22 antiparallel β-strands, the OM also contains a considerable number of smaller, monomeric β-barrel proteins called minor porin proteins, which are composed of 8 or 10 β-strands (Nikaido, 2003; Pages et al., 2008). Although their functions remain largely unknown, these proteins have been implicated in a wide range of physiological processes such as lipid metabolism, cell adhesion, membrane structural stability, as well as stress adaptations (Lin et al., 2002; Hong et al., 2006). OmpW is such a minor porin protein widely distributed in Gram-negative bacteria (**Supplementary Figure S1**). Although its functions remain obscure, it has been implicated in bacterial responses to various antibiotics stresses and environmental stimuli. For instance, OmpW expression was reported to be induced in the presence of minimal inhibitory concentration of tetracycline and ampicillin, in a nalidixic acid-resistant *E. coli* K-12 strain (Xu et al., 2006; Lin et al., 2008), and during the growth on the mucus membrane (Chang et al., 2004). Temperature also modulates the expression of *E. coli* OmpW, suggesting its role in bacterial adaptation to warm-blooded host (Brambilla et al., 2014). In *Salmonella enterica* serovar Typhimurium expression of *ompW* was induced by the presence of methyl viologen (MV) and consequently the protein was suggested to mediate the eﬄux of MV (Gil et al., 2007). However, in another study, its expression was found to be down-regulated by hypochlorous acid and hydrogen peroxide and was suggested to mediate the influx of HOCl and H_2_O_2_ (Morales et al., 2012). In *Vibrio cholerae*, expression of OmpW was found to be affected by a broad range of cultural conditions such as temperature, salinity, and availability of nutrients or oxygen, and consequently was suggested to be involved in the stress adaption of the bacterium (Chang et al., 2004; Nandi et al., 2005; Xu et al., 2006; Gil et al., 2007; Lin et al., 2008). However, no defined physiological functions of OmpW have been reported thus far.

The initial physiologically relevant function of OmpW reported thus far is that it serves as the receptor of Colicin S4, a type of bacterocin produced by certain *E. coli* strains that is lethal to the related strains (Pilsl et al., 1999; Cascales et al., 2007). Recently, *E. coli* OmpW was indicated to participate with small multidrug resistance protein member EmrE to expel quaternary cationic compounds (Beketskaia et al., 2014), and was demonstrated to be required for resistance to phagocytosis (Wu et al., 2013). Bioinformatics studies revealed that OmpW protein is highly conserved in facultative anaerobes including clinically significant pathogens such as: enterohemorrhagic *Escherichia coli* (EHEC), *Salmonella typhimurium*, *Enterobacter cloacae* and *Klebsiella pneumonia*, suggesting potential roles of OmpW in bacterial adaptation or pathogenicity. *Vibrio cholerae* OmpW, which is present in all known *Vibrio* strains, has been found to be highly immunogenic and attracted interests for vaccine development (Jalajakumari and Manning, 1990; Das et al., 1998; Söderblom et al., 2005) for decades. The X-ray crystal structure of *E. coli* OmpW has been resolved and it was shown to form an 8-stranded β-barrel with a long and narrow hydrophobic channel (Hong et al., 2006). Recently, the NMR structure of *E. coli* OmpW has also been determined (Stanczak et al., 2012; Horst et al., 2014). Yet, its expression under ordinary laboratory conditions is low and its regulatory mechanisms and physiological functions remain to be disclosed.

Recent CHIP-chip and CHIP-seq studies of the anaerobic global regulatory systems of FNR and ArcAB revealed that both FNR and ArcA repress the expression of *ompW* under anaerobic glucose fermentative condition (Myers et al., 2013; Park et al., 2013). These findings provide the physiological context to explore the regulation and function of OmpW. Hence, in the current study we systematically investigated the regulation of *ompW* expression and explored its functional relevance to the anaerobic adaptation of bacteria using the facultative bacterium *E. coli* as a paradigm. We show that expression of *ompW* is both activated and repressed by FNR through its direct binding to two atypical binding sites on the *ompW* promoter. While binding of one site causes activation of *ompW*, subsequent binding of the second molecule of FNR to the other site leads to repression of the gene. ArcA also represses *ompW* expression but the regulation is dependent on the simultaneous presence of FNR. Furthermore, expression of *ompW* is subjected to the regulation of several other global regulatory factors that sense and respond to the availabilities of carbon and electron acceptors under anaerobic conditions, such as CRP and NarL. These regulatory patterns combined with the results from molecular docking experiments led us to propose that the previously recognized Colicin S4 receptor protein OmpW plays a role in the transition from aerobic to anaerobic lifestyle of *E. coli*.

## Materials and Methods

*Escherichia coli* DH5α was utilized as the host strain for molecular cloning and plasmids propagation. A BL21 derivative PK22, was used for protein over-expression (Lazazzera et al., 1993). *E. coli* K-12 strain MG1655 served as the parental strain for gene deletions or chromosomal FLAG tagging. Its genomic DNA was utilized as the template for PCR amplification. *E. coli* strains usually were cultured in LB or M9 minimal media. The composition of M9 medium used for gene regulation and physiological studies includes appropriate concentration (w/v) of carbon source (i.e., 0.2% glucose, 0.2% galactose, 0.2% arabinose, or 0.4% glycerol), 0.2% casamino acids (w/v), 1 mM MgSO_4_, 1 mM CaCl_2_, 2.5 mg ml^-1^ ferric ammonium citrate, 2 mg ml^-1^ thiamine and 0.02% ammonium molybdate (w/v). For certain experiments, the medium was also supplemented with 0.4 mM potassium nitrate, 40 mM sodium fumarate, or 40 mM Trimethylamine *N*-oxide (TMAO). Appropriate antibiotics, when required, were supplemented to the media at the following concentrations: Ampicillin (100 μg ml^-1^), Chloramphenicol (20 μg ml^-1^), Kanamycin (25 μg ml^-1^), or Tetracycline (10 μg ml^-1^). Conditions for aerobic, micro-aerobic, and anaerobic culturing of bacteria are as followings. Aerobic: 3 mL of inoculum is cultured in a 15 mL volume culture tube with 220 rpm aeration; Micro-aerobic: 30 mL of inoculum is cultured in a 50 mL volume tube with 14 rpm aeration. The micro-aerobic growth condition in this system is achieved by limited air supply and aeration. The extent of oxygen limitation was recorded by the *promoter-lacZ* activity of the well studied reporter gene *narG* (Kang et al., 2005), which activity in this system is found to be ~50% of its full activity as measured under anaerobic growth condition. Anaerobic: 10^5^–10^7^ cells are inoculated into a screw-capped Pyrex tube filled up with media and are incubated without shaking. Anaerobic growth condition in this system is generated through the following manners: (i) utilization of a screw-capped Pyrex culture tube filled with growth media; (ii) a small initial inoculums (<1/1000 total volume of the media), which can rapidly consume the limited residual O_2_ in the media, and (iii) without aeration supply. The micro-aerobic culturing system described.

### Bacterial Strain Construction

Strains, plasmids and primer sequences described in this work are listed in Supplementary Tables S1–S3. For PCR verification of the constructed strains and plasmids, *FastTaq* (Roche) was used. And for molecular cloning and site-directed mutagenesis, *iProof* (BIO-RAD) was used for the amplification of DNA fragments. Strains containing P*ompW-lacZ* or *ompW*-FLAG fusion on the chromosome of *E. coli* K-12 MG1655 were constructed following the method described by Datsenko and Wanner (2000). To construct P*ompW-lacZ*, promoter region of *ompW* (-215 to +56 relative to *ompW* transcription start site) flanked by XhoI and BamHI at its 5′- and 3′- respectively was amplified by PCR using *E. coli* MG1655 genomic DNA as the template. Following enzyme digestion and purification of the digestion product, the DNA fragment was ligated into pPK7035 (Kang et al., 2005) at the position between the kanamycin resistance gene and *lacZ* such that the expression of *lacZ* is driven by the *ompW* promoter (pPK7035-P*ompW*). Subsequently, a linear DNA fragment containing 36 nt homologous region to *lacI* followed by *kan*-P*ompW*-*lacZ* and ~900 nt homologous region to the downstream of *lacZ* was amplified by PCR using pPK7035-P*ompW* as the template. Following Dpn I digestion to remove the template, the PCR product was purified using illustra^TM^ GFX PCR DNA and Gel Band Purification Kit (GE Healthcare). The purified PCR product was then electroporated into the competent cells of MG1655 transformed with the plasmid pKD46 which contains λ-red recombinase. Desirable colonies were selected on LB plate containing kanamycin and were verified by DNA sequencing.

*PompW*-*lacZ* fusions containing various mutations were constructed by site-directed mutagenesis of pPK7035-P*ompW* followed by PCR amplification of the linear DNA fragment for the pKD46 mediated homologous recombination using the mutated pPK7035-P*ompW* plasmid as the template. This resulted in the strains of AY0234, AY0284, AY0285, AY2010, AY2048, respectively. P*ompW*-*lacZ* fusion was transduced from AY0210 via P1 *vir* into PK4811, PK8281, AY2203, AY2204, AY2206, AY2020 to study the effect of *fnr*, *crp*, *narL*, *narP*, deletion on the transcriptional activity of *PompW-lacZ.* Construction of *ompW*-FLAG was similar to that of *PompW-lacZ* except for the primers used to amplify the linear DNA fragment which contains 50 bp homologous region upstream of the *ompW* stop codon followed by the sequence of FLAG, kanamycin resistance gene, and 50 bp homologous to sequences downstream of *ompW* stop codon. Following the successful construction, the *ompW*-FLAG loci was also transduced from AY0264 to PK4811 via P1 *vir* to study the effect of deletion of *fnr* on the production of OmpW-FLAG. Other strains containing gene deletions were generally constructed by P1 *vir*-mediated transduction from the corresponding Keio collection strains [National BioResource Project (NIG, Japan): *E. coli*]. When necessary, pCP20, a plasmid which encodes FLP recombinase was used to remove the antibiotic resistance marker (Datsenko and Wanner, 2000).

### Site-Directed Mutagenesis

To replace or delete nucleotides in the *ompW* promoter region, site-directed mutagenesis PCR was performed. A pair of complementary mutagenesis primers were designed, containing 45–50 nucleotides with specific mutations in the center, and pAY0201 (**Table 1**) served as the template. Reactions were as below: 5X iProof GC buffer, 2.0 μl; DMSO, 1 μl; 10 mM dNTP mix, 0.4 μl; 5 mM forward and reverse primers 2.0 μl each; template, 10–100 ng; *iProof* (2 units μl^-1^), 0.2 μl; add ddH_2_O to a total volume of 20 μl. The PCR was performed using a thermal cycler C1000 (BIO-RAD). The program was set as follows: 98°C * 3 min; 98°C * 10 s, 50°C * 1 min, 72°C * 5 min, for 18 cycles; 72°C * 10 min. PCR products were subsequently treated with 1 μl *Dpn*I (20 units μl^-1^, NEB) at 37°C for 1 h to remove the remaining template. The resulting products were transformed into *E. coli* DH5α competent cells. Mutations were verified by DNA sequencing.

**Table 1:** Binding affinity of various compounds to OmpW estimated by molecular docking.

Ligands	Estimated free energy of binding
LDAO	-6.17 kcal/mol
Fumarate	-3.91 kcal/mol
TMAO	-1.90 kcal/mol

### β-Galactosidase Assay

*Escherichia coli* strains containing promoter*-lacZ* fusions were inoculated in M9 minimal medium (final cell density as ~10^5^ cells ml^-1^) containing appropriate concentration (w/v) of carbon sources (0.2% glucose, 0.2% arabinose, or 0.4% glycerol), 0.2% CAA (w/v), 1 mM MgSO_4_, 1mM CaCl_2_, 2.5 mg ml^-1^ ferric ammonium citrate, 2 mg ml^-1^ thiamine and 0.02% ammonium molybdate (w/v). 0.4 mM potassium nitrate or 40 mM sodium fumarate was also added as indicated in certain experiments. Cells were grown aerobically by shaking at 250 r.p.m. to an OD_600_~0.3 or anaerobically in screw-capped tubes without aeration to an OD_600_ ~0.2 at 37°C. Chloramphenicol (50 μg ml^-1^) or Tetracycline (20 μg ml^-1^) was then added to terminate cell growth and any further protein synthesis. Cells were placed on ice until assayed as described previously (Miller, 1992). Proper volume of cells were mixed with Z buffer (60 mM Na_2_HPO_4_⋅7H_2_O, 40 mM NaH_2_PO_4_⋅H_2_O, 10 mM KCl, 1 mM MgSO_4_⋅7H_2_O, 50 mM β-mercaptoethanol, pH 7.0) to a total volume of 1 ml and lysed by adding chloroform and SDS. Following thorough mixture and incubation at 28°C water bath for 5 min, 200 μl ortho-Nitrophenyl-β-galactoside (ONPG, 4 mg ml^-1^) was added to initiate the reaction. Upon the development of yellow color, reactions were stopped by adding 500 μl freshly prepared Na_2_CO_3_ solution and vortex. A_420_ and A_550_ of the reaction solution, OD_600_ of cell culture, and time required for color development in each of the reactions were recorded to calculate the β-Galactosidase activity. The activity is expressed in Miller Units as the mean from three independent experiments. Error bars represent the standard deviation.

### Total RNA Isolation

Anaerobic or aerobic cell culture was obtained as described above in β-galactosidase assay. 8 ml anaerobically grown culture of *E. coli* MG1655 was mixed with 1.25 ml ice-cold ethanol/phenol stop solution (5% water-saturated phenol pH4.5 in ethanol) and placed on ice for 10 min before being harvested by centrifugation at 4000 *g* for 9 min at 4°C. After removing supernatant the cell pellet was frozen in liquid nitrogen and stored at -80°C to aid lysis. Cells were lysed by resuspending in 800 μl TE buffer (30 mM Tris⋅Cl, 1 mM EDTA, pH 8.0) containing 1.4 μl 36 kU μl^-1^ lysozyme (Epicentre), and then placed in 64°C water bath for 2 min. After incubation, 88 μl 3M NaOAc (pH 5.2) was added to adjust the pH and ion strength of the lysate solution. Subsequently, acid-phenol/chloroform extraction followed by ethanol precipitation was performed to obtain the total RNA following the manufacture’s instruction. To remove trace amount of genomic DNA contamination, the extracted RNA was subject to DNase I treatment using the turbo DNA Free Kit (Amibion). Absence of genomic DNA contamination was confirmed by PCR using the prepared RNA as template. The quantity of RNA was determined using NanoDrop 2000 (Thermo Scientific).

### Reverse Transcription to obtain cDNA

~1 μg RNA prepared as described above was mixed with 1.5 μl 10 μM gene-specific reverse primer or 1 μl 200 μg μl^-1^ random primers (Promega), and incubated at 70°C for 5 min followed by an ice bath for 5 min. The following components were then added sequentially: 5 μl 5 × RT buffer (Promega), 5 μl 10 mM dNTP mix (Promega), 200 units of MMLV reverse transcriptase (Promega), and 20 units of RNase inhibitor (Roche). The mixture was incubated in 40°C water bath for 1 h and then the reaction was terminated by incubation at 70°C for 15 min. The quantity and purity of cDNA was determined using a NanoDrop 2000 (Thermo Scientific).

### RT-qPCR

The specific primer pairs were designed using on-line tool^1^. *rrsA* gene of the 16S rRNA was chosen as the normalizing gene. RT-qPCR was performed with each specific primer pair (qRT-ompW+: CCGTACGTCCAACAGAAGGT, qRT-ompW-: TGCCAGTAATTCCACACCAA) using SYBR Green Mastermix (ABI). The reactions were conducted on an ABI StepOnePlus real-time PCR detection system, and the fluorescence signal of SYBR green intercalation was monitored to quantify the double-stranded DNA product formed in each PCR cycle. Data was analyzed using ΔΔCt method according to the manufacturer’s instruction. Experiments were performed using the cDNA sample obtained from three independent isolates, and error bars represent the standard deviation.

### RNA Ligase-Mediated Rapid Amplification of 5′ cDNA Ends (5′ RLM-RACE)

Total RNA was isolated from *E. coli* MG1655 WT strain as mentioned above. The extracted RNA was first hydrolyzed by Tobacco Acid Pyrophosphatase (TAP) followed by ligation to the 5′ RACE adapter. The resulting RNA was then reverse transcribed to cDNA using reagents supplied in the FirstChoice RLM-RACE Kit (Ambion) following the manufacture’s instruction. The obtained cDNA was then utilized to perform outer (primer: *ompW* 5′race out-: 5′-GTTGGTGGCAGATGATGAACGGTT-3′) and inner (primer: *ompW* 5′race in-: 5′-CCGCTCGAGCGCTGCCAGTAATTCCACACCAATGT-3′) PCR using Fastart Taq (Roche) DNA polymerase. The PCR products were gel band purified and cloned into BamHI and XhoI sites of pPK7035 (Kang et al., 2005) followed by DNA sequencing. The first nucleotide being sequenced following the 5′ RACE adapter sequence was determined as the transcription start site.

### EB-Stained EMSA

Ethidium bromide (EB)-stained EMSA was performed according to a previous report (Nishino and Yamaguchi, 2002) with slight modifications. DNA fragments of 100–250 bp *ompW* promoter regions were prepared by PCR and purified using illustra^TM^ GFX PCR DNA and Gel Band Purification Kit (GE Healthcare). The binding reaction was conducted in a 10 μl reaction system containing variable amount of protein and DNA fragment in the binding buffer (20 mM Tris-HCl [pH 8.0], 50 mM NaCl, 1 mM EDTA, 1 mM β-mercaptoethanol, and 10% glycerol). 0.2 mM cAMP was added to each reaction when the binding of His_6_-CRP to the *ompW* promoter was examined, and 5 μg of Bovine serum albumin (NEB) mixed with DNA served as a negative control. Following incubation at 37°C for 30 min, the reaction mixtures were loaded onto a 6% poly-acrylamide gel. Electrophoresis was conducted at 120V for 1.5–2 h in an ice-bath. The gel was stained with EB and photographed under UV illumination.

### DIG (Digoxigenin)-Labeled EMSA

Digoxigenin-labeled EMSA (Shan et al., 2012) was performed to detect the binding of His_6_-NarL or phosphorylated His_6_-NarL (His_6_-NarL-P) to *ompW* promoter. Phosphorylated protein was obtained following a previous protocol with slight modification (Lukat et al., 1992): 1 μg of purified His_6_-NarL was incubated with 25 mM Tris-HCl, 0.05 mM EDTA, 25 mM acetyl phosphate, 5% glycerol, and 10 mM MgCl_2_ in 10 μl reaction for 1 h at 30°C. DNA fragment encoding the promoter of *ompW* was purified and labeled using Roche DIG Gel Shift Kit (second generation) as following: the DNA fragment was diluted to 10 ng μl^-1^ with ddH_2_O and mixed sequentially with labeling buffer, CoCl_2_-solution, DIG-ddUTP solution and terminal transferase; the mixture was incubated at 37°C for 15 min before the labeling reaction was stopped with 0.2 mM EDTA. The probe was then purified with illustra^TM^ GFX PCR DNA and Gel Band Purification Kit (GE healthcare) and eluted with ddH_2_O. Each EMSA reaction mixture contained 1 ng of DIG-labeled DNA and appropriate amount of phosphorylated protein as described in figure legends. Five microgram of Bovine serum albumin (NEB) mixed with DNA served as the negative control. The binding reaction, incubation and electrophoresis were performed as described in EB-staining EMSA. Following electrophoresis DNA probes were transferred to positive charged nylon membrane (Roche) in 10X SSC solution (1.5 M NaCl, 150 mM Sodium Citrate), followed by detection using Roche DIG Gel Shift Kit (second generation).

### Measurement of the Production of OmpW-FLAG by Western-Blot

Anaerobic and aerobic cultures of AY0264 and AY0265 were obtained as described in β-galactosidase assay. Eight mililiter culture (O.D. as 0.3) was pelleted by centrifugation at 4000 *g* for 9 min at 4°C and subsequently resuspended in 30 μl BugBuster Protein Extraction Reagent (Novagen) containing 1 mg ml^-1^ lysozyme and 10 U ml^-1^ DNase I to lyse the cells. The mixture was incubated at 37°C for 30 min followed by centrifugation at 16000 *g* for 20 min at 4°C. The supernatant was transferred to another fresh tube. To avoid any insufficient lysis, the pellet was resuspended in 10 μl 4X SDS buffer followed by incubation at 55°C for 25 min. The supernatant was then combined with the 30 μl supernatant from lysis by the BugBuster Protein Extraction Reagent and incubated at 55°C for another 25 min. After centrifugation at 16000 *g* for 1 min, a portion of the protein extract was subject to total protein analysis using the Bio rad Bradford Protein Assay kit. ~ 40 μl supernatant (the exact volume of each of the sample was adjusted based on the total protein content measurement) was loaded onto SDS-PAGE and then subjected to western blot analysis using 1:5000 mono-clonal anti-FLAG (Sigma) primary antibody and anti-mouse IgG secondary antibody (Sigma).

### Purification of His_6_-Tagged Protein

For over-expression of His_6_-tagged FNRD154A, CRP and NarL, overnight cultures of AY2023, AY2030, AY0984 were diluted 1:50 to 50 ml LB containing 20 μg/ml kanamycin, and grown at 37°C with shaking for ~2.5 h (or ~5 h for AY0984) till OD_600_~0.6. 0.5 mM IPTG was then added to the culture to induce the expression of His_6_-tagged protein at 30°C for 4 h. For over-expression of His_6_-tagged Colicin S4, the plasmid p33-S4His containing the DNA sequence encoding Colicin S4 supplied by Prof. Dirk Linke’s group (Arnold et al., 2009) was transformed into *ompW* knock-out strain AY0250. Overnight culture of AY0250/p33-S4His was diluted 1:50 to 50 ml LB containing 100 μg/ml ampicillin, and grown at 37°C with shaking for ~2.5 h till OD_600_~0.6. The expression of His_6_-colicin S4 was then induced by adding 0.2 μg/ml anhydrotetracycline at 30°C for 4 h. Cells overexpressing His_6_-tagged protein were then harvested by centrifugation at 4000 *g* for 9 min at 4°C, and stored at -80°C until needed. Cell pellet was thawn on ice and then resuspended in 5 ml ice-cold lysis buffer [50 mM Tris-HCl buffer pH 7.2, 10% glycerol, 500 mM NaCl, 20 mM imidazole, 0.5 mM DL-Dithiothreitol (DTT), 0.15 mM Phenylmethanesulfonyl fluoride (PMSF)]. Cell suspension was lysed by sonication for 10 min (duty cycle = 60%, output control = 6) using VWR SCIENTIFIC BRANSON SONIFIER 450. The resulting cell lysate was then subjected to ultracentrifuge at 45000 *g* for 30 min at 4°C. The supernatant was mixed with 2 ml Ni Sepharose 6 Fast Flow resin (GE Healthcare) and gently shaken for 1 h at 4°C to allow sufficient binding. The mixture was then poured into a 1.5 cm X 10 cm column and washed with 10 ml binding buffer (50 mM Tris-HCl pH 7.2, 10% glycerol, 500 mM NaCl, 40 mM imidazole). His_6_-tagged protein was eluted with 5 ml elution buffer (50 mM Tris-HCl pH 7.2, 10% glycerol, 500 mM NaCl, 250 mM imidazole). The elution was dialyzed for 3 h in 250 ml dialysis buffer (50 mM Tris-HCl pH 7.2, 10% glycerol, 500 mM DTT, 0.5 mM EDTA) for three times at 4°C to remove imidazole. SDS-PAGE was used to monitor the purification process and analyze the purified protein. The concentration of purified protein was determined using NanoDrop 2000 (Thermo).

### Molecular Docking

The 3D structure of OmpW was obtained from RSCB Protein Data Bank^2^ (PDBID: 2F1T) (Hong et al., 2006), which contains three chains (A, B, C) and an embedded lauryldimethylamine-oxide (LDAO) molecule in the membrane channel. Chain A of 2F1T (2F1TA) was selected for the docking study and the missing residues (21–28, the purple part in **Figure 7A**) in 3D structure were completed as a loop using the SWISS-MODEL online homology modeling server. The structures of LDAO which was present in OmpW crystal, and the potential substrates fumarate and TMAO were generated by ChemBioDraw Ultra (ChemOffice, Cambridge, MA 02140, USA), which were subsequently optimized with the MM2 force filed shipped with ChemBio3D. Then, AutoDock 4.2.5.1 was used to study the binding conformations of LDAO, fumarate, or TMAO to 2F1TA, using the Lamarckian genetic algorithm (LGA) (Fuhrmann et al., 2010). The default search parameters given by AutoDockTools 1.5.6 were adopted and the maximum number of energy evaluations was enlarged to 25 million to thoroughly explore the conformation space of LDAO, fumarate or TMAO. The grid boxes are set to 40 × 46 × 92 points and 68 × 68 × 126 points respectively, with the grid spacing kept at 0.375 Å to cover both the original binding cavity of LDAO and the transmembrane domain of OmpW. Then 50 LGA runs were performed with AutoDock on a small cluster for both grid boxes settings.

### Colicin Rescuing Assay

For the rescuing assay under aerobic condition, *E. coli* MG1655 strain was inoculated (cell density as ~10^5^ cells ml^-1^) in M9 minimal medium containing 0.4% glycerol (w/v) and grown at 37°C with shaking till OD_600_~0.1. Colicin S4 (final concentration as 0.002 μg μl^-1^) and 0–10 mM of fumarate, succinate, or TMAO as indicated in **Figure 8** was then added to the cell culture. Cells were continuously grown aerobically at 37°C and OD_600_ was recorded every 30 min using a SPECTRONIC 20D+ (Thermo Scientific) till the culture reached stationary phase. For the rescue assay under anaerobic conditions, *E. coli* MG1655 strain was inoculated (cell density as ~10^5^ cells ml^-1^) in M9 minimal medium containing 0.4% glycerol (w/v) and 40mM TMAO and grown at 37°C anaerobically till OD_600_~0.1. Colicin S4 (final concentration as 0.002 μg μl^-1^) and 0–10 mM of fumarate as indicated in **Figure 8** was then added to the cell culture. Cells were continuously grown anaerobically at 37°C and OD_600_ was recorded every hour till the culture reached stationary phase.

## Results

### FNR Activates the Expression of *ompW* under Anaerobic Condition

**FIGURE 1:**
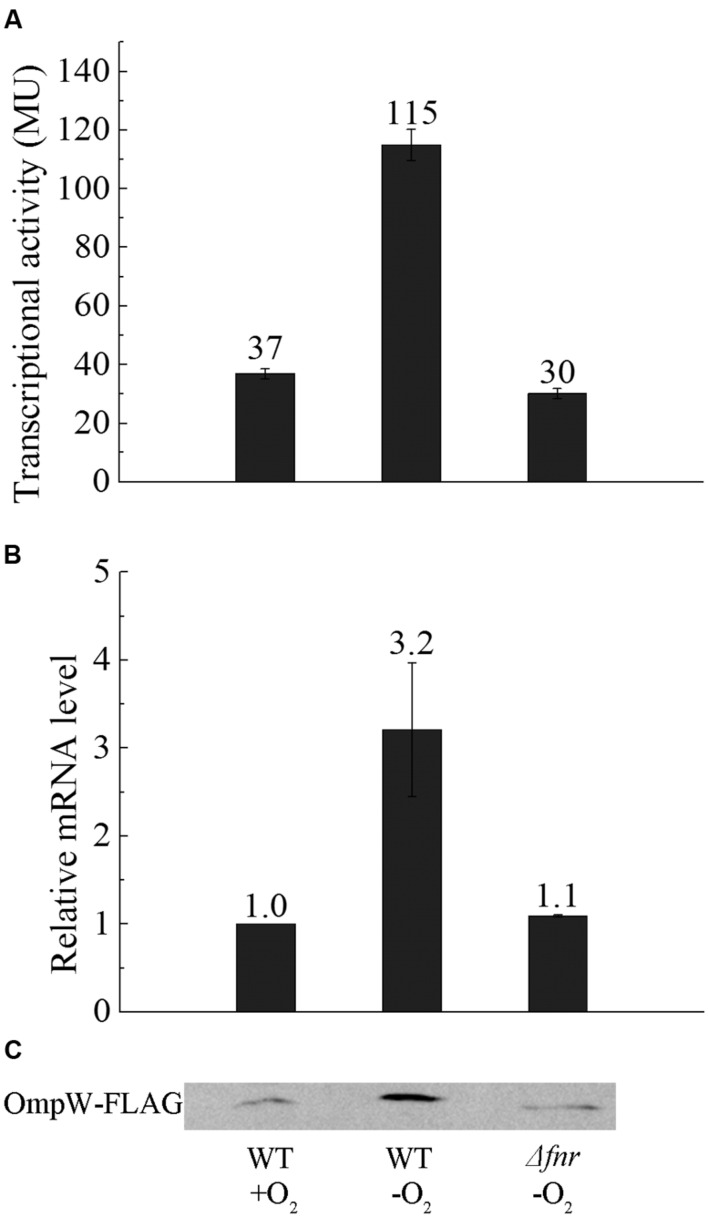
**Expression of OmpW is up-regulated by FNR under anaerobic growth condition in *E. coli*. (A)** β-galactosidase assay of *PompW-lacZ* fusion under aerobic and anaerobic condition and in *Δfnr* strain under anaerobic condition in M9 glucose medium. Transcriptional activity is expressed in miller units (MU), and error bars represent the standard errors of triplicate experiments (*n* = 3). **(B)** Fold changes of the mRNA levels of OmpW in MG1655 WT strain and its *Δfnr* derivative in M9 glucose medium under anaerobic conditions relative to that in MG1655 cultured under aerobic conditions as determined by RT-qPCR. Error bars represent the standard errors of triplicate experiments (*n* = 3). **(C)** Western blot analysis of the production of chromosomal OmpW-FLAG in MG1655 or the isogenic *Δfnr* cells cultured under the same conditions as in β-galactosidase assay.

To investigate whether the expression of *ompW* is indeed directly regulated by FNR as indicated in the genomewide studies by Dufour et al. (2010) and Myers et al. (2013), we constructed chromosomal P*ompW-lacZ* in *E. coli* MG1655 and its isogenic Δ*fnr* strain and examined *in vivo* transcription of *ompW* gene under both aerobic and anaerobic conditions. β-galactosidase assay showed that transcription of P*ompW-lacZ* under anaerobic condition was increased to ~3-fold of that under aerobic conditions, and deletion of *fnr* caused decrease of the transcription to the similar level as that of aerobic condition, suggesting that transcription of *ompW* was activated by FNR under anaerobic condition (**Figure 1A**). To confirm this up-regulation at mRNA and protein level, we performed reverse transcription-quantitative PCR (RT-qPCR) and western blot analysis using the chromosomal FLAG-tagged OmpW strain under identical conditions as in β-galactosidase assay. RT-qPCR and western blot showed that mRNA level and protein level of OmpW under anaerobic condition was also induced to ~3-fold compared with that under aerobic condition, and deletion of *fnr* caused reduction of *ompW* mRNA and production of OmpW-FLAG (**Figures 1B,C**), confirming the anaerobic activation of *ompW* in an FNR-dependent manner.

### Anaerobic Expression of *ompW* Is Modulated by FNR through its Direct Binding to Two Distinctive Sites

**FIGURE 2:**
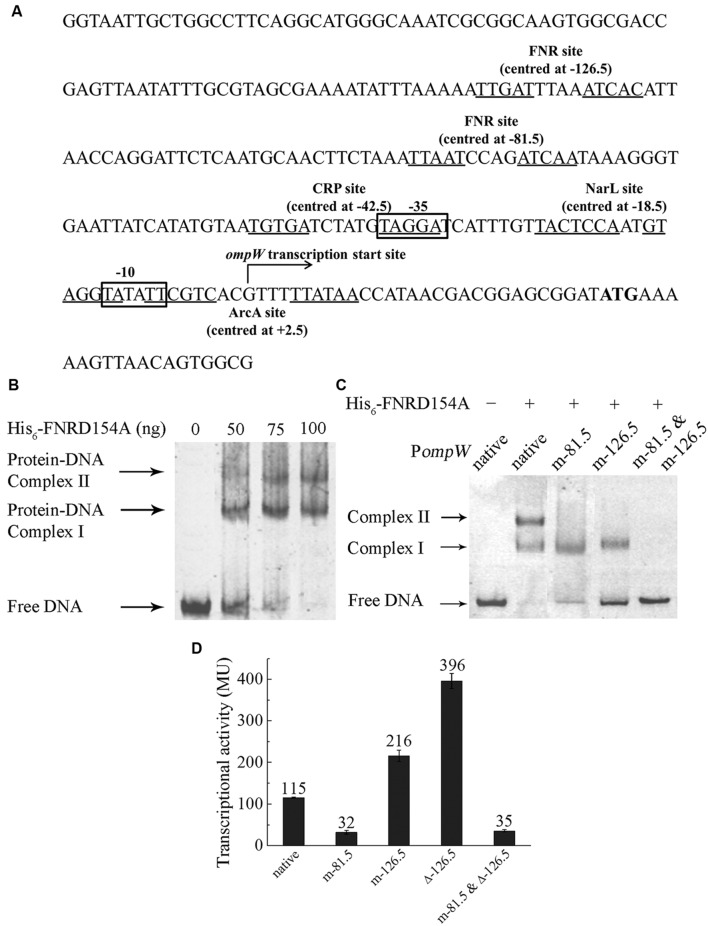
**FNR directly binds to the *ompW* promoter and regulates its expression through its binding to the sites centered at -81.5 and -126.5. (A)** Schematic diagram of the *ompW* promoter region (-215 to +56 bp relative to the transcription start site). ATG start codon is in bold; transcription start site is indicated by the arrow; -10 and -35 elements are boxed; putative binding sites of FNR, ArcA, CRP and NarL are underlined and their binding sites relative to the transcription start site are indicated. **(B)** EMSA of His_6_-FNRD154A to the -215 to +56 bp DNA fragment of the native *ompW* promoter (P*ompW*). **(C)** EMSA of His_6_-FNRD154A with native (-167 to -36 bp fragment relative to the transcription start site) or various of mutant *ompW* promoters. **(D)** Transcriptional activity of P*ompW-lacZ* containing the native promoter, mutation of the -81.5 site (m-81.5), mutation of the -126.5 site (m-126.5), mutation of both sites (m-81.5 and m-126.5), deletion of the -126.5 site (Δ-126.5), and the combination of mutation of the -81.5 site with deletion of the -126.5 site (m-81.5 and Δ-126.5) under anaerobic condition as determined by β-galactosidase activity assay. Error bars represent the standard errors from three independent isolates (*n* = 3).

FNR regulation is mediated through its specific binding to the consensus DNA sequence of “TTGATN_4_ATCAA” in the promoter of its regulated genes (Spiro and Guest, 1990; Khoroshilova et al., 1995; Green et al., 1996). In most of FNR regulated promoters, the binding sites are centered either at -41.5 (Class II FNR-dependent promoter) or -61.5 (Class I FNR-dependent promoter) bp upstream of the transcription start site (Wing et al., 1995; Wing et al., 2000; Green et al., 2001; Korner et al., 2003). To investigate whether FNR directly binds to the promoter of *ompW* and activates its expression, we performed electro-mobility shift assay (EMSA) using the FNRD154A variant which exists as a functional dimer even under aerobic conditions (Lazazzera et al., 1993). EB staining of the EMSA reactions revealed that FNR indeed directly bound to the promoter region of *ompW* as a shifted band corresponding to the protein-DNA complex was observed upon the addition of FNRD154A protein (**Figure 2B**). Unexpectedly, an additional super-shifted band was observed in EMSA at higher concentration of FNRD154A protein implying the presence of a second FNR binding site on the promoter of *ompW* (**Figure 2B**). To confirm this, we first performed bioinformatics analysis and found that indeed two putative FNR binding sites are present in P*ompW*: in addition to the site centered at -126.5 bp (TTGATTTAAATCAC) upstream of *ompW* transcription start site which was identified by Myers et al. (2013) using CHIP-seq, a binding site centered at -81.5 bp (TTAATCCAGATCAA) which bears 9 out of 10 FNR consensus site was also identified (**Figure 2A**). In order to verify whether FNR indeed binds to these two sites and regulates the expression of *ompW*, we mutated either or both of the sites and examined the effect of these mutations on the binding of FNR to *ompW* promoter. To disrupt the -81.5 site, we changed the last four nucleotides of the site from “TCAA” to “CTGG”. To disrupt the -126.5 site, the third nucleotide “G” in the first half of the binding site “TTGAT” was changed to “A” and the last four nucleotides “TCAC” were changed to “CTGG”. As shown in **Figure 2C**, two retarded bands corresponding to the DNA-protein complexes were observed in the EMSA assay of FNRD154A with the native promoter. However, only one retarded band was observed in the EMSA of FNR with either mutated -81.5 site or mutated -126.5 site (**Figure 2C**). Furthermore, promoter DNA containing mutations on both sites failed to form any complex with FNRD154A (**Figure 2C**). These results confirmed that FNR can directly bind to both -81.5 and at -126.5 sites in the promoter region of *ompW*.

To investigate the role of FNR binding to each of the sites on the anaerobic regulation of *ompW in vivo*, we mutated each of the two sites in the P*ompW*-*lacZ* fusion and compared their transcription activity with that of the native promoter. As shown in **Figure 1**, mutation of the -81.5 site caused ~3 fold reduction of the transcription of *ompW* to the similar level as that of native promoter under aerobic condition or that in *Δfnr* strain under anaerobic condition, suggesting that binding of FNR to the -81.5 site was responsible for the FNR dependent activation of *ompW* gene. Surprisingly, mutation of the -126.5 site led to ~2-fold increase of the transcription of P*ompW-lacZ*, suggesting that FNR binding of this site caused repression of *ompW* transcription under anaerobic condition (**Figure 2D**), consistent with the finding from the CHIP-seq experiment by Myers et al. (2013). To confirm this result, we deleted the entire 14 bp of the -126.5 site in P*ompW-lacZ* and found that transcriptional activity of the resulting Promoter-*lacZ* fusion was even further increased to the level of ~3-fold higher compared to that of native promoter (**Figure 2D**), suggesting that binding of FNR to the -126.5 site indeed led to repression of *ompW* expression. The fact that deletion of the -126.5 site (Δ-126.5) led to a greater extent of derepression of *ompW* transcription than that of m-126.5 suggested that nucleotides change in m-126.5 was not sufficient to completely abolish the binding of FNR to this site. Furthermore, when mutation of the -81.5 site was combined with deletion of the -126.5 site, transcriptional activity of the resulting P*ompW-lacZ* was similar as that of the -81.5 site mutation alone (**Figure 2D**), suggesting that FNR binding of the -126.5 site was dependent on its primary occupancy of the -81.5 site. Taken together, these results suggested that FNR can bind to two distinctive sites on the promoter region of *ompW* and regulate its expression. While binding of the -81.5 site activated the expression of *ompW*, binding of the -126.5 site led to repression of *ompW* expression, and this repression was dependent on the initial occupancy of FNR on the -81.5 site.

Because the locations of both of the sites differ from the positions of the conventional Class I or Class II FNR-dependent promoters which are centered at -61.5 or -41.5 respectively, we wondered whether the transcription start site of *ompW* was not annotated properly previously. To elucidate this, we performed 5′ RLM-RACE to examine the transcription start site of *ompW*. 5′ RLM-RACE result showed a single amplified PCR product (data not shown) and sequencing of this fragment revealed that the first nucleotide being transcribed was “G” located 29 bp upstream of the ATG start codon (**Figure 2A**), the identical site as annotated previously in a genome-wide study (Mendoza-Vargas et al., 2009). This result suggested that FNR bound to two unconventional sites at the *ompW* promoter and regulated its expression.

### Coordinated Regulation of *ompW* via the Binding of FNR to Two Distinctive Sites Led to the Maximal Expression of the Gene under Microaerobic Condition

The pattern of FNR dependent regulation of *ompW* expression is uncommon, yet intriguing. Two additional promoters have been previously shown to contain multiple FNR binding sites and binding of FNR to different sites also led to different effect on the transcription of the genes, such as *cydAB* (encoding cytochrome *bd*) and *focA-pflB* (encoding pyruvate formate lyase) (Sawers, 1993; Govantes et al., 2000). The coordinated regulation of these genes by FNR through its binding to different sites has been shown to be necessary for maximal expression of the genes under micro-aerobic condition and their functions during the transition from aerobic to the anaerobic lifestyle of *E. coli*. This promoted us to investigate whether *ompW* also displays maximal expression under micro-aerobic condition and whether it also functions to facilitate the transition of *E. coli* from aerobic to anaerobic condition.

**FIGURE 3:**
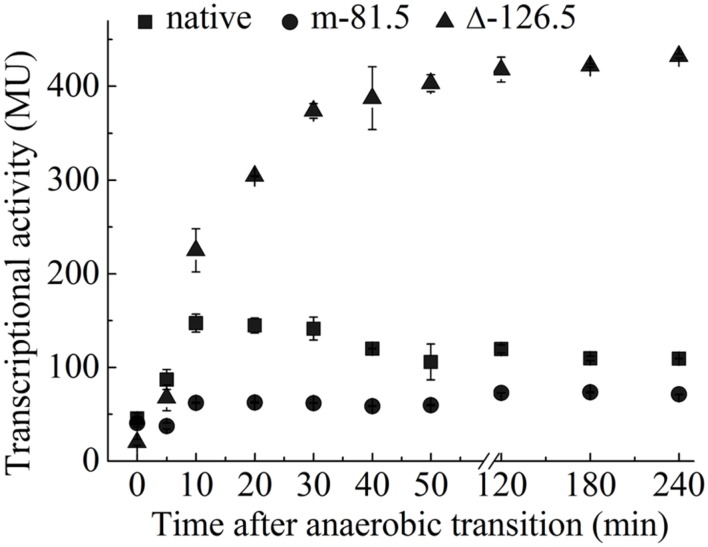
**Coordinated regulation of *ompW* via the binding of FNR to two distinctive sites leads to its maximal expression under microaerobic condition.** Transcriptional activity of P*ompW-lacZ* or the promoter containing mutation of the -81.5 site or deletion of the -126.5 site in M9 glucose medium following the transition from aerobic to the anaerobic growth. Error bars represent the standard errors of triplicate experiments (*n* = 3).

To test this, we examined the transcription of P*ompW-lacZ* at different time points following the transition from aerobic to anaerobic growth. As shown in **Figure 3**, transcription of *ompW* increased following the transition to anaerobic condition and achieved the maximal expression level after 10 min of the transition. Interestingly, transcription of *ompW* was then gradually decreased after 30 min time point. This pattern is consistent with our speculation that FNR binds to the two distinctive sites of *ompW* promoter and gene repression following the FNR binding of the second site (centered at -126.5) was dependent on its binding of the first site (centered at -81.5) which activated *ompW* expression (**Figure 3**). Consistent with this notion, transcription of P*ompW-lacZ* which contains mutation of the -81.5 site was not activated after the transition to anaerobic growth and remained at a very low level during the time course tested; whereas transcription from P*ompW-lacZ* which contained deletion of the -126.5 site was increased rapidly to the level that is even higher than that of native promoter and maintained at this level without obvious reduction for 4 h following the transition (**Figure 3**). These results confirmed the maximal expression of *ompW* under micro-aerobic condition during the transition from aerobic to anaerobic condition and the distinctive effect of the binding of FNR to the two sites, -81.5 and -126.5, on the expression of *ompW*. We also constructed *ΔompW* strain and performed competition assay to examine whether the regulated expression of *ompW* contributes to the competitive advantage or fitness of *E. coli* during the transition from aerobic to anaerobic growth. Growth competition index was calculated based on the colony forming units (CFU) as following: mutant_anaerobic_/parent_anaerobic_: mutant_aerobic_/parent_aerobic;_ mutant_microaerobic_/parent_microaerobic_: mutant_aerobic_/parent_aerobic._ We found *ΔompW* cells displayed a significantly lower competition index (0.25) relative to the WT under microaerobic condition than under anaerobic condition (0.45) (data not shown), suggesting a role of OmpW in the microaerobic environment which occurred during the transition from aerobic to anaerobic lifestyle of the bacterium.

### Transcription of *ompW* Is also Subject to Repression by ArcA

**FIGURE 4:**
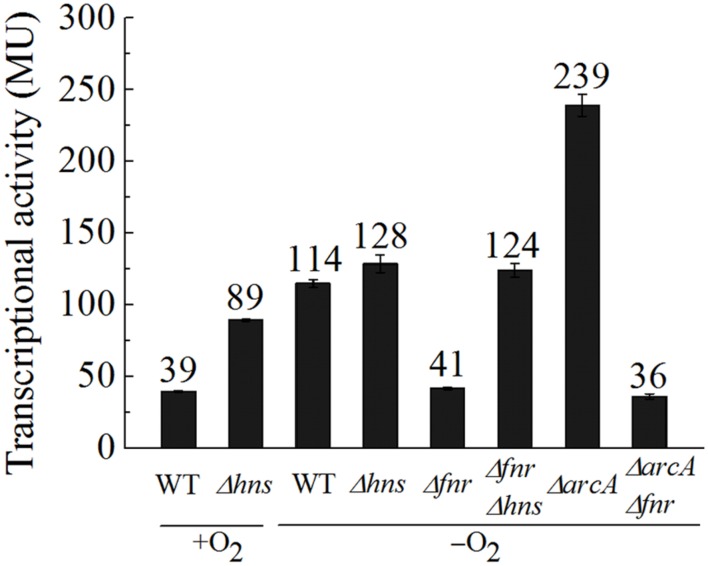
**FNR antagonizes H-NS mediated repression of *ompW* under anaerobic conditions and ArcA represses the transcription of *ompW* in an FNR dependent manner.** β-galactosidase activities of P*ompW-lacZ* in WT, *Δhns*, *Δfnr*, *ΔarcA*, *Δfnr ΔarcA*, and *Δfnr Δhns* strains under aerobic or anaerobic conditions. Error bars represent the standard errors from three independent isolates (*n* = 3).

The fact that deletion of the -126.5 site led to a level of *ompW* transcription that is even higher than that of the WT promoter at all time points following the transition to the anaerobic condition suggested that an additional repression factor may exist and its effect is dependent on the FNR binding to the -126.5 site. Since Park et al. (2013) have indicated that the other anaerobic global regular ArcA also represses *ompW* transcription by CHIP-seq experiment, we then tested the effect of Δ*arcA* on the transcription of P*ompW*-*lacZ* under anaerobic conditions. As shown in **Figure 4**, indeed, Δ*arcA* caused elevated transcription of *ompW*. However, Δ*arcA*Δ*fnr* double deletion resulted in a similar level of *ompW* transcription as that of Δ*fnr* alone, suggesting that ArcA repression of *ompW* transcription was dependent on the presence of FNR, presumably its sequential binding to the -81.5 and -126.5 sites as illustrated above. EMSA also confirmed the direct binding of ArcA to the *ompW* promoter (data not shown).

We next examined how the expression of *ompW* was controlled at low level under aerobic condition. Myers et al. (2013) have showed that many FNR binding sites are masked by the nucleoid-associated proteins H-NS and its paralog StpA. Thus, we tested whether transcription of *ompW* is also repressed by H-NS under aerobic condition. As shown in **Figure 4**, Δ*hns* caused elevation of the transcription of P*ompW*-*lacZ* under aerobic condition, suggesting that aerobic expression of *ompW* was indeed repressed by H-NS. Interestingly, Δ*hns* did not lead to increased transcription of P*ompW*-*lacZ* under anaerobic condition and Δ*fnr*Δ*hns* double deletion caused increased transcription of *ompW* comparing with that of Δ*fnr* alone, suggesting that FNR antagonized the H-NS mediated repression under anaerobic condition. Two different antagnization mechanisms of H-NS mediated gene repression has been described: the antagonist binds to a region previously bound by H-NS; or a region adjacent to the H-NS sites (Dillon and Dorman, 2010). Both mechanisms led to the displacement of H-NS from the promoter DNA and consequently de-repression of the gene transcription. Since we have identified the FNR activation site (centered at -81.5) which differs from the H-NS signature sites (repeated AT rich region), the antagonization of FNR on H-NS is likely through its binding to a separate site, rather than the competitive binding of the same site of H-NS. Taken together, our data suggested that under aerobic conditions, transcription of *ompW* was primarily repressed by the nucleoid-associated protein H-NS. When the bacterium transits from aerobic to anaerobic growth, FNR antagonized the H-NS mediated repression through its binding to the -81.5 site. With the increasing concentration of FNR, the second FNR molecule bound to the -126.5 site and repressed its expression. Binding of FNR to the *ompW* promoter also allowed ArcA to bind to the *ompW* promoter and further repressed *ompW* expression under anaerobic condition.

### Expression of *ompW* Is Subjected to Catabolite Repression via CRP

**FIGURE 5:**
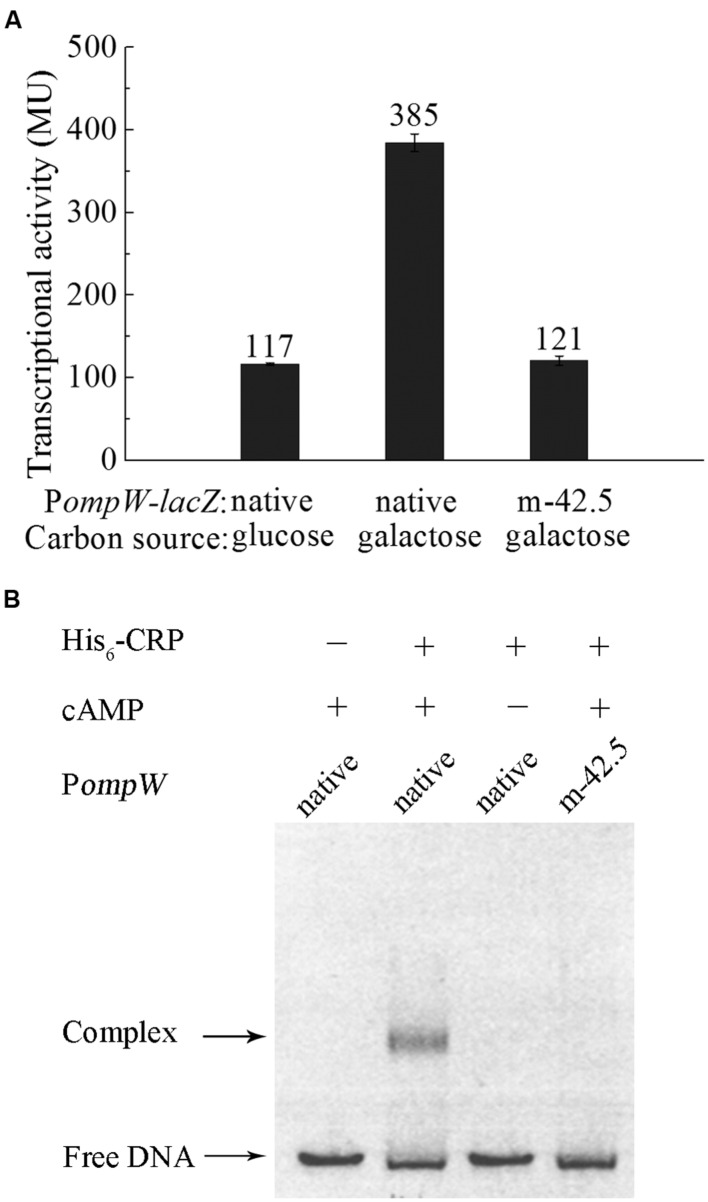
**CRP mediates the catabolite repression of *ompW* through its direct binding to the site centered at -42.5. (A)** Transcriptional activity of the native P*ompW-lacZ* or the promoter containing mutation of the -42.5 site grown in M9 media supplemented with glucose or galactose under anaerobic condition. Error bars represent the standard errors from three independent isolates (*n* = 3). **(B)** EMSA of His_6_-CRP with the native or mutated P*ompW*. The DNA fragment used in the assay encompasses the -120 to -3 bp of P*ompW* in the presence or absence of 0.2 mM cAMP in the reaction as indicated.

To further investigate the role of OmpW in the anaerobic adaptation of *E. coli*, we examined the expression of *ompW* gene in response to other physiologically relevant signals during anaerobic metabolism of the bacterium, such as different carbon sources and electron acceptors. As shown in **Figure 5A**, transcription of P*ompW-lacZ* was elevated in the presence of galactose in comparison with that of glucose M9 medium. Since catabolism of carbon sources other than glucose is primarily controlled by the global catabolite repression protein CRP, we next examined whether transcription of *ompW* is also subject to the activation by CRP.

Bioinformatics search revealed a putative CRP binding site, “5′-TGTGATCTATGTAGGA,” centered at -42.5 bp of the *ompW* promoter (**Figure 2A**) which represents a well conserved CRP site (Gaston et al., 1990; Ushida and Aiba, 1990). To examine whether CRP indeed binds to this site and activates the expression of *ompW*, we disrupted the binding site by deletion of the first five nucleotides “TGTGA” and measured the transcription of this mutated P*ompW*-*lacZ* fusion (m-42.5) in M9 galactose medium. As shown in **Figure 5A**, disruption of this site caused significant decrease of transcription from the P*ompW*-*lacZ* fusion in M9 galactose medium to the level that is similar as that of native promoter in M9 glucose medium, suggesting that the site “5′-TGTGATCTATGTAGGA” was responsible for the elevated transcription in M9 galactose medium. To confirm this *in vitro*, we performed EMSA using purified His-CRP protein and P*ompW* DNA fragments. As shown in **Figure 5B**, EMSA demonstrated that native P*ompW* formed a retarded complex with CRP and mutation of the -42.5 site was sufficient to abolish the binding of CRP to the promoter DNA (**Figure 5B**). It is noteworthy that binding of CRP to the native *ompW* promoter in EMSA was dependent on the presence of cAMP (**Figure 5B**), indicating that the binding of CRP to *ompW* promoter was specific and functionally relevant. Together these results confirmed that CRP directly binds to *ompW* promoter and activates its expression in the absence of the preferred carbon source glucose.

### Nitrate Represses *ompW* Expression through the NarXL Two-Component System

**FIGURE 6:**
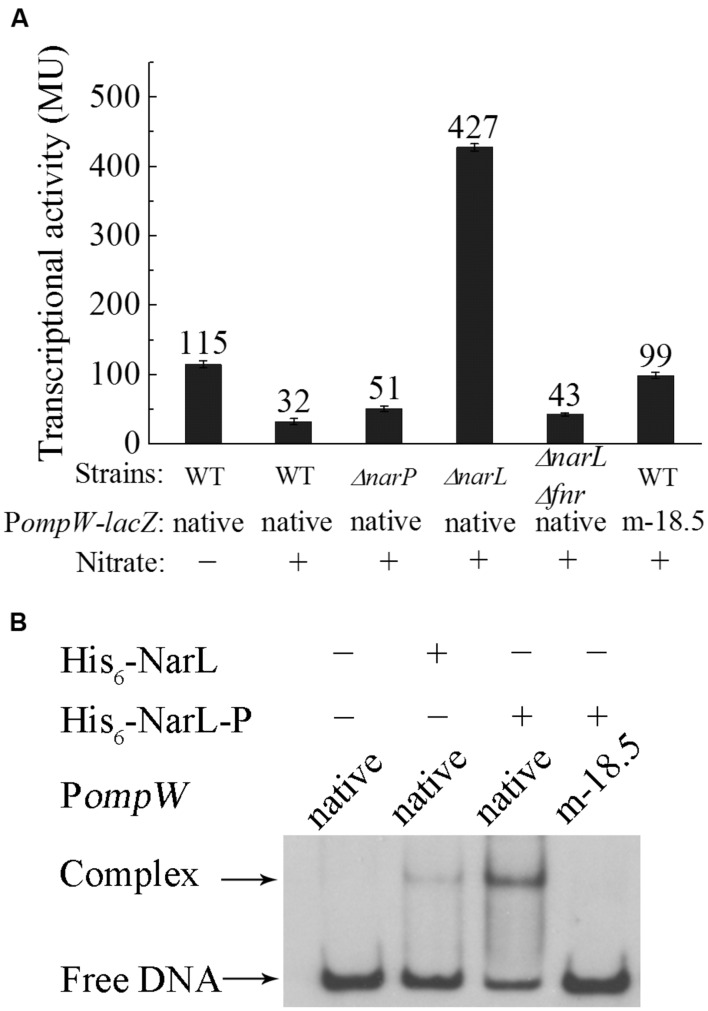
**Nitrate represses the expression of *ompW* in a NarL dependent manner. (A)** Transcriptional activity of native P*ompW-lacZ* or promoter containing mutation of the putative NarL binding site (m-18.5) in WT, *ΔnarP*, *ΔnarL* or *ΔnarPΔnarL* strains grown in M9 glucose media with or without nitrate under anaerobic condition. Error bars represent the standard errors of triplicate experiments (*n* = 3). **(B)** DIG-labeled EMSA of His_6_-NarL with the native or mutated P*ompW*. DNA fragment used in the assay is the same as in **Figure 5**. One nanogram of DIG-labeled native promoter DNA or that containing mutation of the putative NarL binding site (m-18.5) was incubated with (+) or without (-) 100 ng of protein in a 10 μl binding reaction. His_6_-NarL-P indicates phosphorylated His_6_-NarL.

Depending on the presence or absence of alternative electron acceptors, facultative bacteria either respire or ferment under anaerobic conditions. Since anaerobic respiration conserves energy with higher efficiency than fermentation, this mode of metabolism is preferred under anaerobic condition when alternative electron acceptors are present. Among the various electron acceptors which can be utilized by *E. coli*, nitrate is preferred to all other compounds owing to its significantly positive standard redox potential and consequently the highest energy yield (Goh et al., 2005). Thus, presence of nitrate has a profound effect on the metabolism of *E. coli* under anaerobic conditions and it represses a broad range of genes involved in the utilization of other alternative electron acceptors (Constantinidou et al., 2006). Hence, we also measured the expression of *ompW* in the presence of nitrate in glucose M9 medium. It was shown that transcription of P*ompW-lacZ* decreased significantly in the presence of nitrate (**Figure 6A**), suggesting that nitrate repressed the transcription of *ompW*. It is known that nitrate regulation is mediated by the NarX-NarL and NarQ-NarP two-component systems (Darwin et al., 1996) in which NarX and NarQ are the sensor kinases and NarL and NarP are the response regulators respectively. They recognize a consensus heptameric DNA binding sequence of “TACYYMT” (where Y = C or T and M = A or C) present in the promoter regions of the target genes and regulate their expression (Tyson et al., 1993, 1994). To examine how the presence of nitrate led to repression of *ompW*, we constructed *ΔnarP*, *ΔnarL* and *ΔnarPΔnarL* mutants and measured the transcription of P*ompW-lacZ* in these strains. As shown in **Figure 6A**, deletion of *narP* did not have a significant effect on the transcription of P*ompW-lacZ* in the presence of nitrate. However, deletion of *narL* led to significant de-repression of the transcription of P*ompW-lacZ*, and *narP narL* double deletion had a similar effect as that of *narL* single deletion (**Figure 6A**), suggesting NarL was responsible for the transcriptional repression of *ompW* in the presence of nitrate. Furthermore, NarL mediated repression was found to be dependent on FNR, since *fnr narL* double deletion resulted in a similar transcription activity of P*ompW-lacZ* as that of *Δfnr* alone and *ΔnarL* no longer had de-repression effect in the *Δfnr* strain background. On the other hand, transcription of P*ompW-lacZ* was not affected by nitrate or *narL* deletion under aerobic growth condition (data not shown). Although the exceptionally high β-galactosidase activity of P*ompW-lacZ* resulted from *ΔnarL* was ambiguous, nonetheless, these results suggested that expression of *ompW* was subject to nitrate repression in a NarXL dependent manner under anaerobic condition.

To further confirm NarL-dependent repression of *ompW*, we analyzed the sequence of its promoter region and identified a potential NarL binding site centered at -18.5 bp (TACTCCAATGTAGGTA) upstream of the transcription start site (**Figure 2A**). Mutation of the first three nucleotides of the site from “TAC” to “CAT” relieved the nitrate repression of P*ompW-lacZ* (**Figure 6A**). We next performed EMSA to detect the direct binding of NarL to the P*ompW* DNA fragment. As shown in **Figure 6B**, a retarded band corresponding to the protein-DNA complex formed by phosphorylated NarL (His_6_-NarL-P) and native P*ompW* was observed and His_6_-NarL-P displayed a significantly higher binding efficiency than that of unphohphorylated His_6_-NarL, suggesting the specific binding of the phosphorylated NarL to the *ompW* promoter. However, DNA fragment containing mutation of the -18.5 site failed to form a complex with His_6_-NarL-P (**Figure 6B**), indicating that NarL bound to the site centered at -18.5 on P*ompW* and repressed its expression, presumably by interfering with the binding of RNAP to the promoter given the close proximity of this site to the -10 and -35 elements of the promoter.

### Molecular Docking Suggested the Binding of Fumarate to OmpW

**FIGURE 7:**
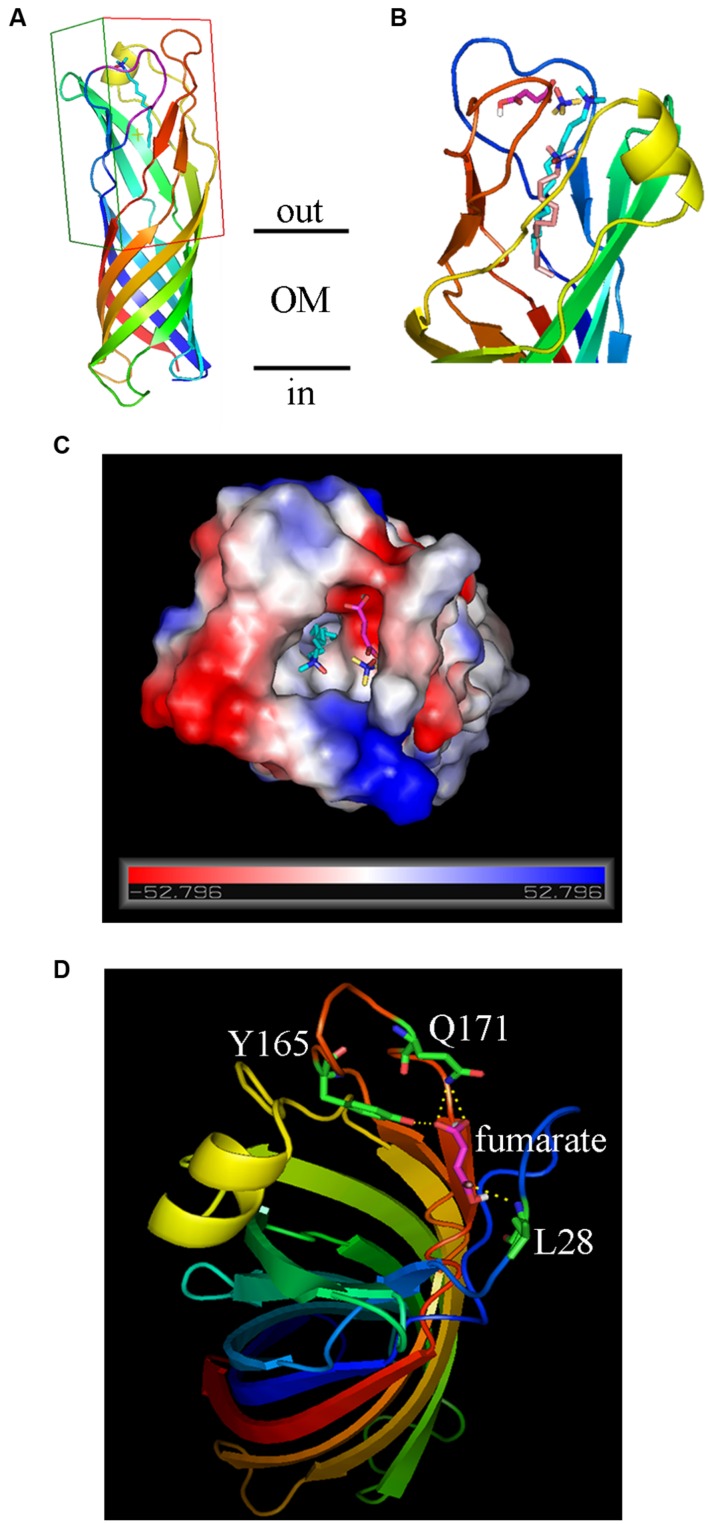
**Molecular docking of the binding of fumarate to OmpW. (A)** Ribbon diagrams of OmpW structure viewed from the side, the AA residues are rainbow colored from blue (N-terminus) to red (C-terminus). The original LDAO ligand is colored in cyan. The grid box for AutoDock study is outlined. **(B)** Ribbon diagrams, 90° rotated relative to **(A)**, showing the predicted binding positions of LDA (light pink) compared with its native position (cyan) as present in the original 3D crystal structure of OmpW. The positions of fumarate (magentas) and TMAO (yellow) are also indicated. **(C)** View of OmpW from the extracellular side, showing the positions of fumarate (magentas) and TMAO (yellow) and LDA (cyan). The electrostatic potential surface of OmpW is plotted and colored according to the surface charge (red for negative, gray for neutral and blue for positive). **(D)** Ribbon diagrams showing that fumarate binding to OmpW could be stabilized by L28, Q171, and Y165.

In bacteria, there are often strong correlations between how transcription of the gene is regulated and the physiological roles of the encoded proteins (Cole, 2012). The fact that transcription of *ompW* is subject to tight controls by the availability of O_2_, carbon sources, and the anaerobically preferable electron acceptor nitrate suggested that OmpW may be involved in the carbon and energy metabolism of the bacterium during anaerobiosis. Interestingly, a previous proteomic study suggested that OmpW directly interacts with fumarate reductase Frd (Huang et al., 2006) which is the key enzyme involved in anaerobic respiration of fumarate. This led us to speculate that perhaps the function of OmpW is relevant to the metabolism of fumarate, the only intermediate of central metabolic pathway (TCA cycle) that can also act as an alternative electron acceptor in anaerobic respiration. To test this, we performed molecular docking to examine the binding of fumarate to OmpW. The 3D structure of OmpW was obtained from the RSCB Protein Data Bank which contained an embedded detergent molecule LDAO. To perform molecular docking, a region encompassed by residues 21–28 which was missed from the original structure was first completed as a loop (**Figure 7A**) using the SWISS-MODEL online homology modeling server. Since the detergent molecule LDAO was present and bond to OmpW in the original 3D structure of OmpW, we first conducted molecular docking of LDAO to OmpW using the in-house LGA algorithm developed by us. LDAO was predicted to bind OmpW with an estimated free energy of binding as -6.17 kcal/mol (**Table 1**) by the algorithm, consistent with the high affinity of LDAO to OmpW pore as indicated in the original 3D structure. Notably, LDAO was also predicted to be present in its native position as in the original 3D structure by our algorithm. Its predicted binding pocket (light pink, **Figure 7B**) was also consistent with its native position (cyan, **Figure 7B**) as present in the original 3D crystal structure except a slight shift of about 2-carbon toward the inner pore of OmpW, which probably was caused by the presence of solvent molecules in the crystal structure and the absence of solvent in the docking prediction. After confirming the feasibility of the docking and the algorithm, binding of fumarate to OmpW was analyzed and it was shown to bind to a side pocket of OmpW that differs from that of LDAO (**Figure 7C**) with an estimated free energy of -3.91 kcal/mol (**Table 1**). Binding of fumarate to this pocket was predicted to be stabilized by L28, Q171, and Y165 (**Figure 7D**). To test whether this binding is specific, we examined the binding of another alternative electron acceptor of anaerobic respiration, trimethylamine *N*-oxide (TMAO), with OmpW and found that TMAO was not predicted to bind to OmpW, since only -1.9 kcal/mol of free energy of binding is predicted by the algorithm. These results suggested that OmpW might be able to bind fumarate and serve as a specific receptor of fumarate.

### Fumarate Is Capable of Rescuing OmpW Mediated Colicin S4 Killing of *E. coli*

**FIGURE 8:**
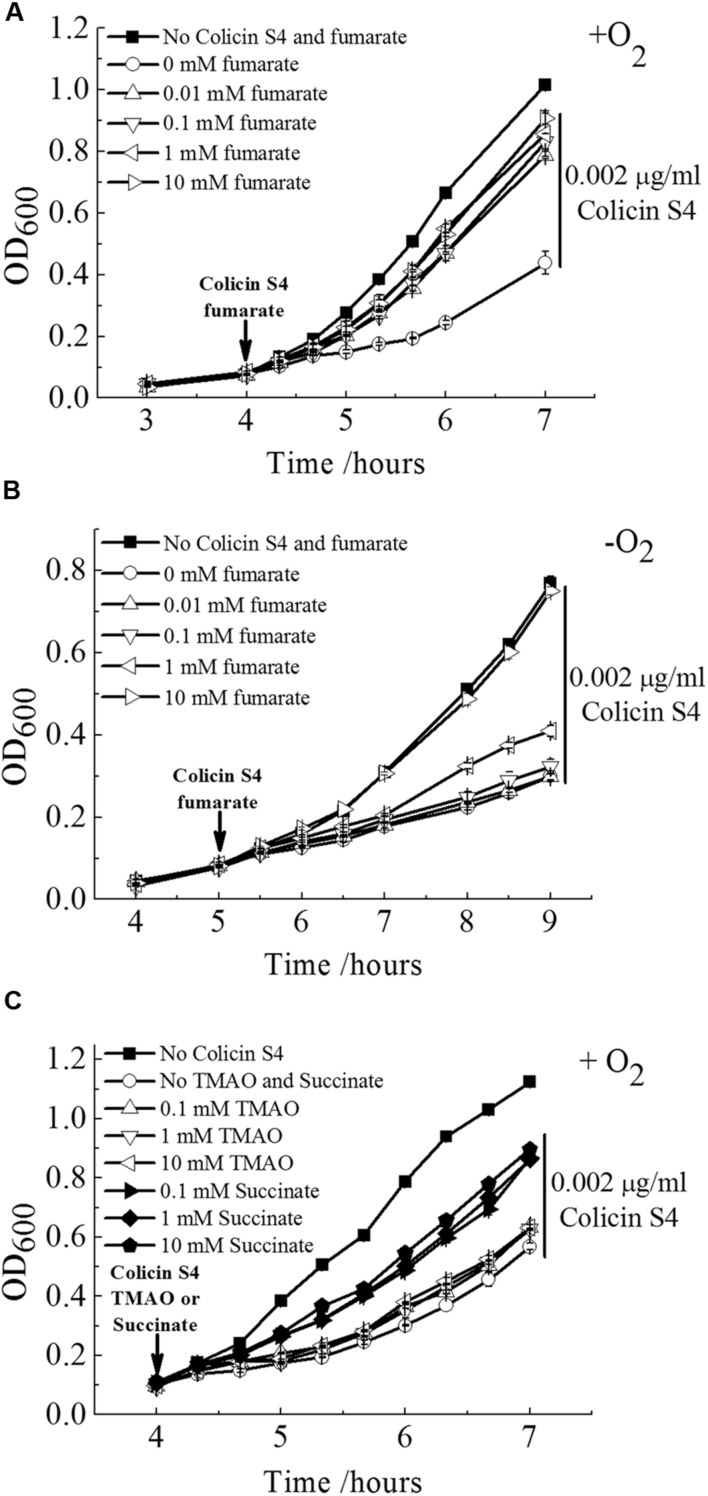
**Fumarate and succinate can rescue Colicin S4 mediated killing of *E. coli*.** Rescue of Colicin S4 mediated growth inhibition of *E. coli* MG1655 cells by fumarate in a concentration dependent manner under aerobic **(A)** and anaerobic **(B)** condition. **(C)** Colicin S4 mediated growth inhibition of *E. coli* MG1655 cells can also be rescued by succinate but not TMAO. Rescue assay was conducted as in **(A)**. Error bars represent the standard errors of triplicate experiments (*n* = 3).

To test our speculation of the physiologically relevant function of OmpW to bind fumarate experimentally, we performed growth rescue assay. It has been known that OmpW serves as the receptor of Colicin S4 during its mediated killing of *E. coli* cells (Pilsl et al., 1999). If OmpW also serves as a fumarate receptor and can bind fumarate with high affinity, we speculate that presence of fumarate will block the binding of Colicin S4 to OmpW and consequently can rescue *E. coli* cells from Colicin S4 mediated killing. Indeed, this method has been used extensively to examine the functions of OM protein receptors since it is common for Colicins to utilize the nutrient receptors located on the OM of the bacterium for its entry. Examples include Colicins A and E1–E9 which utilize vitamin B12 transporter BtuB; Colicin K which binds to the nucleoside transporter Tsx; Colicin V which interacts with the OM porin protein OmpA; and Colicin M which utilizes ferrichrome receptor FhuA and Colicins B and D which recognizes the ferric enterobactin receptor FepA (Cascales et al., 2007). In all these cases, addition of the corresponding natural ligands can protect the bacterium from the specific Colicins mediated killing (Cascales et al., 2007). Thus, to test whether the Colicin S4 receptor OmpW indeed is capable of binding fumarate, we examined whether addition of fumarate can rescue the growth of *E. coli* in the presence of Colicin S4. As shown in **Figures 8A,B**, presence of a very low concentration of purified Colicin S4 (0.002 μg/ml) significantly inhibited the growth of *E. coli* under both aerobic and anaerobic conditions, and addition of fumarate with various concentrations indeed can rescue the Colicin S4 mediated growth inhibition of *E. coli*. Interestingly, it requires higher concentration of fumarate to rescue the Colicin S4 mediated killing under anaerobic than under aerobic conditions, consistent with the higher expression level of OmpW under anaerobic conditions. Since fumarate belongs to a class of metabolites called C_4_-dicarboxylates, we also tested the effect of another C_4_-dicarboxylate, succinate, on the growth of *E. coli* in the presence of Colicin S4. As shown in **Figure 8C**, succinate can partially rescue the growth inhibition of *E. coli* by Colicin S4. However, another alternative electron acceptor TMAO which showed poor or no binding to OmpW in molecular docking is not able to rescue the growth inhibition caused by Colicin S4. These results together suggested that OmpW might be involved in the binding and/or utilization of C_4_-dicarboxylates in *E. coli* during the transition from aerobic to anaerobic lifestyle of *E. coli*.

## Discussion

In recent decades, genome-wide analyses such as microarray, CHIP-chip, and CHIP-seq have allowed identification of global gene expression changes in response to specific physiological stresses and/or the activation of specific transcription regulators. However, a full understanding of bacterial physiology relies on the elucidation of the functions of each of the genes which expression is altered in response to the stimuli. OmpW has been identified as a core regulon of the anaerobic global transcription regulator FNR (Dufour et al., 2010) and its expression has been found to be repressed by both FNR and the other anaerobic global regulator ArcA by CHIP-chip and CHIP-seq analyses (Myers et al., 2013; Park et al., 2013). However, the physiological implication of this anaerobic regulation and its cellular functions of *OmpW* remain obscure. Expression of *Salmonella ompW* has been reported to be up-regulated by MarA and SoxS in response to menadione and down-regulated by ArcA in response to hypochlorous acid and hydrogen peroxide (Gil et al., 2007; Morales et al., 2012; Collao et al., 2013). However, these scenarios are not physiologically relevant. Here, we substantiated the FNR and ArcA mediated repression of *ompW* in *E. coli* shown in the genome-wide analyses and identified an additional FNR binding site (-81.5 site) which is responsible for its activation. We also revealed the regulation of *ompW* by CRP and NarL which responds to the availability of carbon sources and electron acceptors in the growth environment respectively, and the repression of *ompW* expression by the nucleoid-associate protein H-NS under aerobic conditions. On the basis of these regulatory mechanisms, molecular docking and the growth rescue assay of Colicin S4 mediated killing of *E. coli* suggested a role of OmpW in the binding of fumarate and other C_4_-dicarboxylates in responses to the availability of O_2_, carbon sources, and electron acceptors.

**FIGURE 9:**
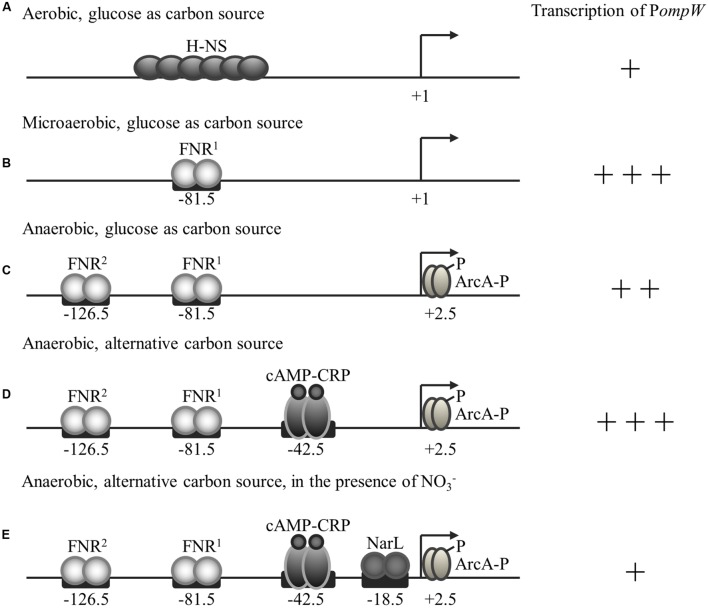
**Regulation of *ompW* expression by H-NS, FNR, ArcA, CRP, and NarL in response to the availability of O_2_, glucose, and nitrate.** Transcriptional activity of *ompW*, its transcription start site, transcription factors and their binding sites are indicated. **(A)** Under aerobic conditions, transcription of *ompW* is primarily repressed by the nucleoid-associated protein H-NS. **(B)** When oxygen concentration starts to reduce during the transition from aerobic to anaerobic growth, FNR first binds to the -81.5 site and activates *ompW* expression by antagonizing the H-NS mediated repression. **(C)** When oxygen concentration continues to reduce and the concentration of active FNR increases, the second FNR molecule binds to the -126.5 site and represses its expression. FNR binding of the *ompW* promoter also allows ArcA binding and further repression of *ompW* expression. **(D)** When the preferable carbon source glucose is absent, CRP is activated and binds to the *ompW* promoter at the site centered at -42.5 to enhance the expression of *ompW* gene. **(E)** When the preferable electron acceptor nitrate is present, NarL is activated and it represses the transcription of *ompW* through its binding with the site centered at -18.5. The number of “+” represent the relative intensity of *ompW* transcription.

Fumarate is a key metabolic intermediate in the TCA cycle and can serve as an alternative electron acceptor during anaerobic respiration. As an intermediate in the TCA cycle, fumarate is oxidized to malate under the catalysis of fumarase Fum. When serving as an alternative electron acceptor of anaerobic respiration, fumarate is reduced by the fumarate reductase Frd to yield succinate. Notably, fumarate is the only intermediate in the central metabolic pathway that can also serve as an electron acceptor in anaerobic respiration. During the transition from aerobic to the anaerobic metabolism, TCA cycle enzymes which function primarily in carbon oxidation are generally repressed, whereas the enzymes that are involved in anaerobic respiration of alternative electron acceptors in the absence of the preferred electron acceptor nitrate are activated. That is, the flux of TCA cycle intermediates is expected to be reduced while the flux of the alternative electron acceptors needs to be increased during the transition to the anaerobic lifestyle. The requirement of this metabolic adaptation probably explains why the transcription of *ompW* was first activated by FNR through its binding to the -81.5 site and subsequently repressed through binding of the second FNR molecule to the -126.5 site, and further repressed by ArcA which functions primarily to repress TCA cycle enzymes. Unlike *fum* and *frd*, which are located in different operons and consequently are controlled independently by different regulators, OmpW as a membrane protein that is capable of binding fumarate and potentially involved in its metabolism is located in a separate location and its expression is driven by a single promoter. This perhaps explains the fact that expression of *ompW* is subject to both activation and repression by a series of the global regulators involved in anaerobic carbon and energy metabolism. Among them, since FNR directly senses the absence of O_2_ molecule and regulates global gene expression involved in the anaerobic lifestyle of *E. coli*, it acts as the primary regulator of *ompW*. Other transcription factors, such as ArcA (senses redox potential of the cell), CRP (senses the availability of alternative carbon sources), and NarL (senses the availability of respiratory electron acceptor nitrate) are the secondary regulators that modulate *ompW* expression in response to the availability and status of energy, carbon, and electron acceptors in the anaerobiosis of the bacterium. The relationship and roles of these regulators on *ompW* expression are supported by our genetic studies. A model to explain this regulation is summarized in **Figure 9**.

Notably, this type of coordinated regulation by the global regulators FNR, ArcA and H-NS is also observed in the case of *cydAB* operon encoding cytochrome *d* oxidase which also displays maximal expression under microaerobic condition during the transition of bacteria from aerobic to anaerobic growth. Interestingly, FNR binding sites on *cyd* promoter, as well as other 15 similar promoters as identified by Myers et al. (2013) in CHIP-seq analysis, were all broadly distributed rather than being located at the traditional FNR binding site centered either at -41.5 or -61.5 bp. Interestingly, those genes were also suggested to have maximal expression under microaerobic conditions (Myers et al., 2013). These observations further support the regulatory mechanisms and physiological functions of OmpW during the transition from the aerobic to the anaerobic lifestyle of *E. coli*.

The OM porin proteins play an important role in the permeability of Gram-negative bacteria which dictate the entry of both nutrients and toxic compounds, such as antibiotics. This is because the OM of Gram-negative bacteria is generally non-permeable to both hydrophilic and hydrophobic molecules owing to the presence of lipopolysaccharide within the outer leaflet of the OM. To facilitate the entry of nutrients into bacteria, bacteria express OM porins (OMP) which form hydrophilic pores and channels in the hydrophobic lipid bilayer of the Gram-negative bacteria OM. The type and expression levels of these porins play a critical role in the selective acquisition of nutrients or toxic compounds in the environment (Nikaido, 2003; Pages et al., 2008). Regulated expression of these proteins represents an important strategy for bacterial survival and adaptation to several of growth environments, especially those in their ecological niches and human host (Lin et al., 2002; De la Cruz and Calva, 2010). Fumarate and other C_4_-dicarboxylates such as L-malate and succinate, are common products of plant and bacterial metabolism. They serve as the major carbon sources of *rhizobia* which form symbiosis with plants (Teramoto et al., 2008; Nisbet et al., 2009; Scheu et al., 2010; Valentini et al., 2011; Pajor et al., 2013). Fumarate is the only metabolic intermediate known to be able to serve as an electron acceptor in anaerobic respiration, and succinate can be reduced from fumarate as catabolic end products by bacteria. Although conceivably the flux of these substances through the OM of Gram-negative bacteria can be achieved by the major porin proteins without the assistance of a receptor, it is likely that a specific receptor is required under the circumstances when this class of nutrients is present at very low level and consequently the transport mode of facilitated diffusion is required which relies on the specific receptors. It is also likely that a specific receptor is needed under the circumstance of C_4_-dicarboxylates serving as the major carbon source of the bacteria such as *rhizobia*. As a matter of fact, not only is *ompW* gene widely distributed in various Gram-negative bacteria, its promoter region also displays certain degree of similarity, especially the FNR (-81.5 site) and CRP sites in several clinically significant species, such as *S. typhimurium*, *Y. pestis*, *K. pneumoniae*, and *E. cloacae* (**Supplementary Figure S2**). All these strongly support a conserved role of OmpW in the cellular fumarate metabolism in Gram-negative bacteria.

An open question is how OmpW participates in the cellular utilization of fumarate. We have attempted to answer this question by extending the grid box in molecular docking to cover the entire trans-membrane part of OmpW (data not shown). However, the docking results showed no further evidence for the transport or flux process. The X-ray crystal structure of OmpW does not show a continuous channel spanning the entire membrane either. However, this could be due to the lack of amino acid residue dynamics in the static X-ray crystallographic structure and the fact that OmpW proein was treated rigid in the docking study. Future work will focus on identifying the proteins interact with OmpW and characterization of OmpW homologues in those species which utilize fumarate as their main carbon source to understand how OmpW facilitate the physiological process of fumarate utilization during the transition of bacteria to their anaerobic lifestyle.

## Author Contributions

AY, MX, JS, and GC designed experiments; MX, YL, and JS performed experiments; MX, YL, and AY prepared the manuscript.

## Conflict of Interest Statement

The authors declare that the research was conducted in the absence of any commercial or financial relationships that could be construed as a potential conflict of interest.

## Footnotes

1 http://frodo.wi.mit.edu/primer3/

2 http://www.rscb.org/

## Supplementary Material

FIGURE S1 **Phylogenetic tree of 71 representative OmpW amino acid sequences constructed based on Maximum Likelihood (ML) method using MEGA with 100 times of bootstrap test for each node**. A database of OmpW protein sequences was made using OmpW (NP_415772.1) of *Escherichia coli* str. K-12 substr. MG1655 as reference to blast against the NCBI GenBank. Seventy one representative OmpW sequences of each genera in all OmpW database was selected to construct phylogenetic tree using Maximum Likelihood criteria in MEGA (Tamura et al., 2013) with 100 times of bootstrap test for each node.

FIGURE S2 **ClustalW alignment of *ompW* promoter region (-250 to -1 bp upstream of ATG)**. The sequences from the following bacterial species are aligned: *E. coli*, *Escherichia coli*; *S. typhimurium*, *Salmonella typhimurium*; *Y. pestis*, *Yersinia pestis*; *K. pneumoniae*, *Klebsiella pneumonia*; *E. cloacae*, *Enterobacter cloacae*. Well conserved motifs corresponding to the binding sites of several global transcriptional regulators are shown.
