# Supplementary figures and images for: Transcriptional Regulation of the Outer Membrane Porin Gene ompW Reveals its Physiological Role during the Transition from the Aerobic to the Anaerobic Lifestyle of Escherichia coli
Source: Front Microbiol. 2016 May 31;7:799. doi: 10.3389/fmicb.2016.00799 (PMC4886647; doi:10.3389/fmicb.2016.00799)

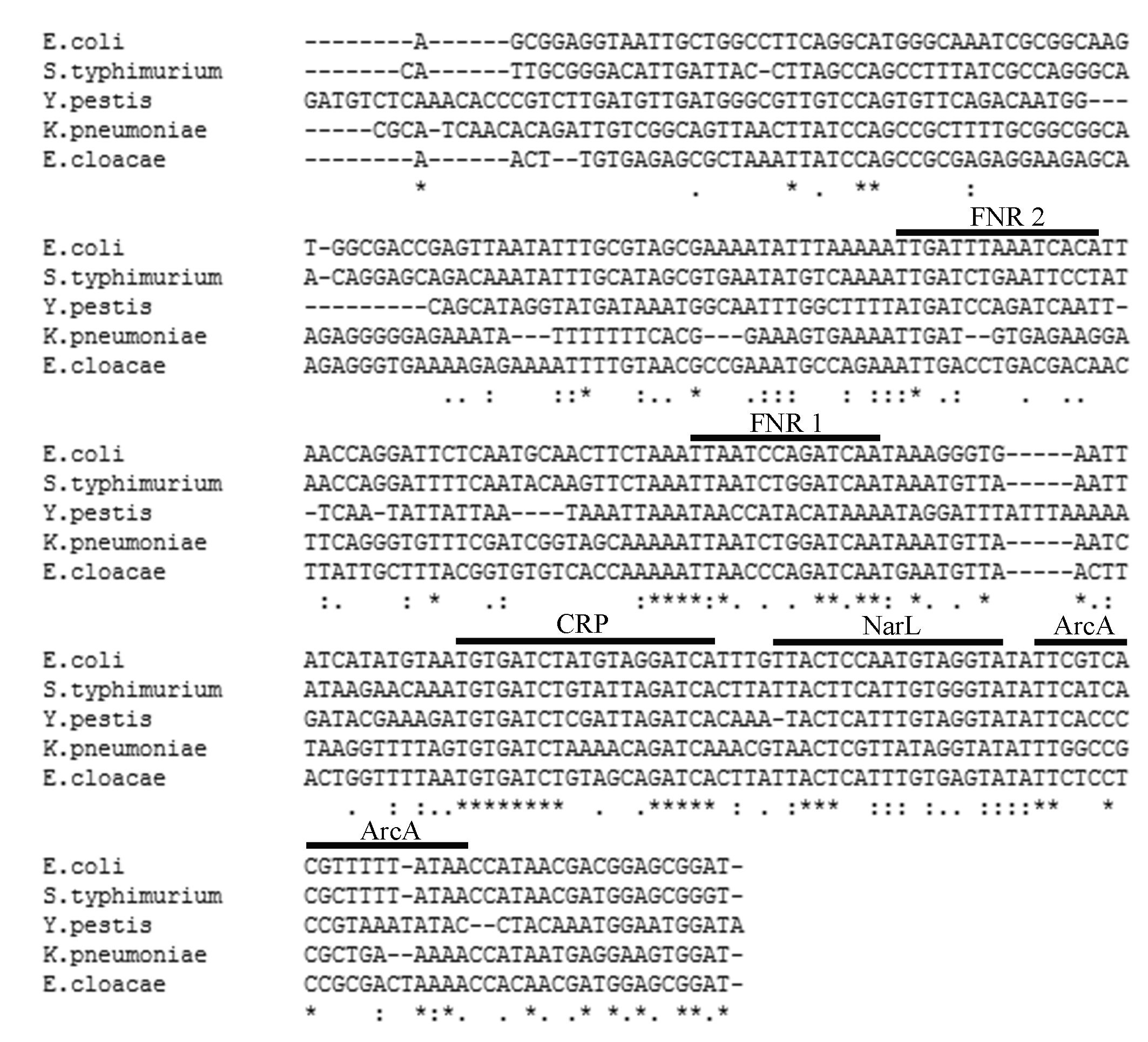

Supplement: FIGURE S2 — ClustalW alignment of ompW promoter region (-250 to -1 bp upstream of ATG). The sequences from the following bacterial species are aligned: E. coli, Escherichia coli; S. typhimurium, Salmonella typhimurium; Y. pestis, Yersinia pestis; K. pneumoniae, Klebsiella pneumonia; E. cloacae, Enterobacter cloacae. Well conserved motifs corresponding to the binding sites of several global transcriptional regulators are shown. [file Image_2.TIF]
