# Supplementary material for: Transcriptional Regulation of the Outer Membrane Porin Gene ompW Reveals its Physiological Role during the Transition from the Aerobic to the Anaerobic Lifestyle of Escherichia coli
Source: Front Microbiol. 2016 May 31;7:799. doi: 10.3389/fmicb.2016.00799 (PMC4886647; doi:10.3389/fmicb.2016.00799)
Supplement: Supplementary file 3 [file Table_1.DOCX]

Table S1. Strains used in this work

| Strain/Plasmid | Relevant genotype | Source |
| --- | --- | --- |
| MG1655 | λ- F- *rph*-1 | Laboratory stock |
| PK4811 | MG165  5 *ΔfnrΩSpr Smr* | Gift from P. Kiley |
| BL21 | F-, ompT, hsdS (rB-mB-), gal, dcm (DE3) | Laboratory stock |
| PK22 | BL21 (DE153) *Δcrp*-bs990 *rpsL* *Δfnr*ΩSp^r^/Sm^r^ *zcj*-*3061*::Tn*10* | Gift from P. Kiley |
| PK8281 | MG1655 *crp::cat* | Gift from P. Kiley |
| AY0210 | MG1655 P*ompW*-*lacZ* | This study |
| AY0213 | PK4811 P*ompW*-*lacZ* | This study |
| AY0234 | MG1655 P*ompW*(-81.5 TTAATN_4_ACTGG)-*lacZ* | This study |
| AY0249 | MG1655 *ompW::cat* | This study |
| AY0250 | MG1655 *ΔompW* | This study |
| AY0264 | MG1655 *ompW*-FLAG | This study |
| AY0265 | PK4811 *ompW*-FLAG | This study |
| AY0284 | MG1655 P*ompW*(-126.5 TTAATN_4_ACTGG)-*lacZ* | This study |
| AY0285 | MG1655 P*ompW*( -81.5 and -126.5 TTAATN_4_ACTGG)-*lacZ* | This study |
| AY2054 | PK8281 P*ompW-lacZ* | This study |
| AY2048 | MG1655 P*ompW*(-42.5 ΔTGTGA)-*lacZ* | This study |
| AY2204 | MG1655 *ΔnarP* | This study |
| AY2203 | MG1655 *ΔnarL* | This study |
| AY2206 | MG1655 *ΔnarPΔnarL* | This study |
| AY0295 | AY2204 P*ompW-lacZ* | This study |
| AY0296 | AY2203 P*ompW-lacZ* | This study |
| AY2002 | AY2206 P*ompW-lacZ* | This study |
| AY2010 | MG1655 P*ompW*(-18.5 CAT/TAC)-lacZ | This study |
| AY2021 | AY2020 P*ompW-lacZ* | This study |
| AY2049 | AY2020 P*ompW*(-81.5 TTAATN_4_ACTGG)-*lacZ* | This study |
| AY2023 | BL21 pET28a-*narL* | This study |
| AY2030 | BL21 pET28a-*crp* | This study |
| AY0984 | PK22 pET28a-*fnr*D154A | This study |
