# Supplementary material for: Transcriptional Regulation of the Outer Membrane Porin Gene ompW Reveals its Physiological Role during the Transition from the Aerobic to the Anaerobic Lifestyle of Escherichia coli
Source: Front Microbiol. 2016 May 31;7:799. doi: 10.3389/fmicb.2016.00799 (PMC4886647; doi:10.3389/fmicb.2016.00799)
Supplement: Supplementary file 4 [file Table_2.DOCX]

Table S2. Plasmids used in this work

| Plasmid | Relevant genotype | Source |
| --- | --- | --- |
| pPK7035 | Kn^r^ gene from pHP45Ω and BamHI-NdeI fragment from pRS1553 into pBR322 | Gift from P. Kiley |
| pKD46 | Phage λ *gam*-*bet*-*exo* genes under P*araB* control | ([17](#_ENREF_17)) |
| pET28a | Expression vector with hexa-histidine tag | TaKaRa |
| p33-S4His | Expression vector with His_6_-tagged colicin S4 | Gift from D. Linke |
| pAY0201 | P*ompW* cloned into XhoI and BamHI sites of pPK7035 | This study |
| pAY0270 | pAY0201 with the -81.5 site of P*ompW* mutated to TTAATN_4_ACTGG | This study |
| pAY0276 | pAY0201 with site -126.5 of P*ompW* mutated to TTAATN_4_ACTGG | This study |
| pAY0278 | pAY0201 with site -81.5 and -126.5 of P*ompW* mutated to TTAATN_4_ACTGG | This study |
| pAY2009 | pAY0201 with site -18.5 of P*ompW* mutated | This study |
| pAY2222 | *narL* cloned into BamHI and XhoI sites of pET28a | This study |
| pAY0668 | *crp* cloned into BamHI and HindIII sites of pET28a | This study |
| pAY0980 | *fnr* with D154A mutation cloned into NdeI and XbaI sites of pET28a | This study |
