# Supplementary material for: Transcriptional Regulation of the Outer Membrane Porin Gene ompW Reveals its Physiological Role during the Transition from the Aerobic to the Anaerobic Lifestyle of Escherichia coli
Source: Front Microbiol. 2016 May 31;7:799. doi: 10.3389/fmicb.2016.00799 (PMC4886647; doi:10.3389/fmicb.2016.00799)
Supplement: Supplementary file 5 [file Table_3.DOCX]

Table S3. Primers used in this work

| Primer name | Sequence |
| --- | --- |
|  |  |
| XhoI-PompW+ | CCGCTCGAGGGTAATTGCTGGCC TTCAGG |
| BamHI-PompW- | CGCGGATCCCAAAGCCGCCACTGTTAACTT |
| pPK7035+ | TACTAAGCTGATCCGGTGGA |
| pPK7035- | GCCAGTGAATCCGTAATCAT |
| Kan+ | GATTGAACAAGATGGATTGCAC |
| Kan- | TCAGAAGAACTCGTCAAGAAGG |
| 5'-lacI-Kn | GGCACGACAGGTTTCCCGACTGGAAAGCGGGCAGTGAGCCGGATCAATTCCCCTGCTC |
| 3'-lacZ-corr | CGTTTCACCCTGCCATAAAG |
| lacI upstream+ | CTGTTGCCCGTCTCACTG |
| lacZ downstream- | CCGCACGATAGAGATTCGG |
| PompW-81.5m+ | AACTTCTAAATTAATCCAGACTGGTAAAGGGTGAATTATCATAT |
| PompW-81.5m- | ATATGATAATTCACCCTTTACCAGTCTGGATTAATTTAGAAGTT |
| ompW-f-lcml | CAATGTAGGTATATTCGTCACGTTTTTATAACCATAACGACGGAGCGGATCTCGAGCCGATCATATTCAATAAC |
| ompW-r-lcml: | TACCATGTCCTTATTGACCCCGTATATTACGGGGTCGTTTTTGTGCGGAAGACCTCGAGGACTAGTGAACCTC |
| PompW-126.5m+ | AGCGAAAATATTTAAAAATTAATCCAGACTGGATTAACCAGGATTCT |
| PompW-126.5m- | AGAATCCTGGTTAATCCAGTCTGGATTAATTTTTAAATATTTTCGCT |
| PompW-42.5del+ | GGGTGAATTATCATATGTAACTATGTAGGATCATTTGTTA |
| PompW-42.5del- | TAACAAATGATCCTACATAGTTACATATGATAATTCACCC |
| qRT-rrsA+ | CGGTGGAGCATGTGGTTTAA |
| qRT-rrsA- | GAAAACTTCCGTGGATGTCAAGA |
| qRT-ompW+ | CCGTACGTCCAACAGAAGGT |
| qRT-ompW- | TGCCAGTAATTCCACACCAA |
| ompW 5'race in- | CCGCTCGAGCGCTGCCAGTAATTCCACACCAATGT |
| ompW 5'race out- | GTTGGTGGCAGATGATGAACGGTT |
| PompW-18.5m+ | ATCTATGTAGGATCATTTGTCATTCCAATGTAGGTATATTCGT |
| PompW-18.5m- | ACGAATATACCTACATTGGAATGACAAATGATCCTACATAGAT |
| NdeI-fnr+ | GGGAATTCCATATGATCCCGGAAAAGCGAATTATA |
| XbaI-fnr- | GCTCTAGAGGCAACGTTACGCGTATGA |
| BamHI-narL+ | CGCGGATCCATGAGTAATCAGGAACCGGC |
| XhoI-narL- | CCGCTCGAGTCAGAAAATGCGCTCCTG |
| BamHI-crp+ | CGCGGATCCATGGTGCTTGGCAAACCGCA |
| HindIII-crp- | CCCAAGCTTACGAGTGCCGTAAACGACGA |
